# How Does Endometriosis Lead to Ovarian Cancer? The Molecular Mechanism of Endometriosis-Associated Ovarian Cancer Development

**DOI:** 10.3390/cancers13061439

**Published:** 2021-03-22

**Authors:** Nozomi Yachida, Kosuke Yoshihara, Manako Yamaguchi, Kazuaki Suda, Ryo Tamura, Takayuki Enomoto

**Affiliations:** Department of Obstetrics and Gynecology, Niigata University Graduate School of Medical and Dental Sciences, Niigata 951-8510, Japan; nyachida@med.niigata-u.ac.jp (N.Y.); manako0131@med.niigata-u.ac.jp (M.Y.); sudakazuaki@med.niigata-u.ac.jp (K.S.); ryo-h19@med.niigata-u.ac.jp (R.T.); enomoto@med.niigata-u.ac.jp (T.E.)

**Keywords:** endometrium, endometriosis, ovarian cancer, genomic linkage

## Abstract

**Simple Summary:**

Driver gene mutations have been identified in not only various types of endometriosis which are considered the origin of endometriosis-associated ovarian cancer but also the normal endometrium which is considered the origin of endometriosis. We focused on genomic linkage from normal endometrium to ovarian endometriosis and endometriosis-associated ovarian cancer (EAOC) and summarized the current knowledge of the commonality and differentiation of genomic features in the uterine endometrium, endometriosis, and EAOC. In addition, we have proposed molecular mechanism of ovarian carcinogenesis from the normal endometrium via endometriosis based on genomic alterations. This review is expected to contribute to research for the prevention of endometriosis and EAOC.

**Abstract:**

Numerous epidemiological and histopathological studies support the notion that clear cell and endometrioid carcinomas derive from ovarian endometriosis. Accordingly, these histologic types are referred to as “endometriosis-associated ovarian cancer” (EAOC). Although the uterine endometrium is also considered an origin of endometriosis, the molecular mechanism involved in transformation of the uterine endometrium to EAOC via ovarian endometriosis has not yet been clarified. Recent studies based on high-throughput sequencing technology have revealed that cancer-associated gene mutations frequently identified in EAOC may exist in the normal uterine endometrial epithelium and ovarian endometriotic epithelium. The continuum of genomic alterations from the uterine endometrium to endometriosis and EAOC has been described, though the significance of cancer-associated gene mutations in the uterine endometrium or endometriosis remains unclear. In this review, we summarize current knowledge regarding the molecular characteristics of the uterine endometrium, endometriosis, and EAOC and discuss the molecular mechanism of cancer development from the normal endometrium through endometriosis in an effort to prevent EAOC.

## 1. Introduction

Epithelial ovarian cancer involves five major histological types: high-grade serous, low-grade serous, clear cell, endometrioid, and mucinous ovarian carcinomas, and the origin of ovarian cancer varies by histological type (the World Health Organization (WHO) classification of tumors of the female genital tract) (http://whobluebooks.iarc.fr/editorialboard/index.php, accessed on: 21 March 2021). Recently, it has been clarified that high-grade serous carcinoma (HGSC) drives serous tubal intraepithelial carcinoma (STIC) [1]. Based on clinicopathological and epidemiological findings, the precursor of ovarian clear cell carcinoma (CCC) and ovarian endometrioid carcinoma is endometriosis [2,3,4,5,6]. Therefore, CCC and endometrioid carcinoma are both called “endometriosis-associated ovarian carcinoma” (EAOC).

Endometriosis, which is defined as the presence of endometrial tissues outside of the uterine cavity, is common in gynecological disease affecting 10–15% of reproductive-age women, causing various symptoms of dysmenorrhea, chronic pelvic pain, and infertility and leading to a reduction in quality of life [7,8]. There are several hypotheses for the development of endometriosis, such as the retrograde menstruation theory, the coelomic metaplasia theory, and the stem cell theory [9]. In 1927, Dr. Sampson proposed that fragments of the menstrual endometrium flow retrograde through the fallopian tubes and then implant at peritoneal surfaces, and this theory is broadly accepted [10]. Another leading theory, the coelomic metaplasia theory, suggests that endometriosis originates from metaplasia of the abdominal peritoneum, as stimulated by hormonal, environmental, or infectious stimuli [11]. Furthermore, it was recently proposed that endometriosis derives from stem/progenitor cells or bone marrow-derived stem cells [9]. To date, numerous studies to elucidate the pathological mechanism of endometriosis have been performed worldwide, though it remains unclear why benign endometriosis causes malignant transformation. Recent genomic studies, including ours [12,13,14], are key to understanding this phenomenon.

In this review, we summarize the current knowledge on the commonality and differentiation of genomic features in the uterine endometrium, endometriosis, and EAOC and describe a proposed molecular mechanism of EAOC development from the normal endometrium via endometriosis based on genomic alterations.

## 2. Genomic Profiling of Endometriosis-Associated Ovarian Cancer

With the improvement of sequencing technology, several studies focusing on genomic features or molecular targets in EAOC have been performed. Table 1 and Table 2 summarize the genomic alterations occurring in CCC [15,16,17,18,19] or endometrioid carcinoma (EC) [20,21,22] based on previously reported results of whole-exome sequencing, respectively. First, the *ARID1A* gene, which encodes a key component of the SWI/SNF complex that plays an important role in chromatin remodeling, is most frequently mutated in EAOC; indeed, 46–70% of CCCs and 19–36% of ECs harbor *ARID1A* mutations. Immunohistochemical analysis has demonstrated that *ARID1A* loss-of-function mutations correlate strongly with loss of ARID1A protein expression, and *ARID1A* loss-of-function mutation is one of the most important driver events in EAOC. In addition to *ARID1A*, *ARID1B* (10–18%) and *SMARCA4* (5–10%), also SWI/SNF complex components, are frequently mutated in CCC, suggesting an important association between aberrant chromatin remodeling and carcinogenesis in CCC.

In general, activation of the PI3K/AKT/mTOR pathway plays a key role in the malignant transformation of tumors and their growth, proliferation, and metastasis [23], and EAOC often harbors gene alterations in multiple components of the pathway. For example, *PIK3CA* mutation is found in 40–51% of CCCs and 27–43% of ECs. *PTEN* is also mutated in 5% of CCCs and 29% of ECs (Table 1), and loss of PTEN expression has been detected in 12–40% of CCCs [24,25,26] and 35–38% of ECs [26,27]. The correlation between loss of ARID1A expression and activation of the PI3K/AKT pathway in CCC has been reported [25,28]. Statistically, *PIK3CA* mutations were more frequently detected in tumors that showed loss of ARID1A expression than in tumors that strongly expressed ARID1A (46% vs. 17%, *p* = 0.013) [28]. In another report, loss of ARID1A expression was more frequent in CCC cases with (54%) than in those without (30%) PI3K/AKT pathway activation (*p* = 0.046) [25]. Several genomic studies based on next-generation sequencing data have demonstrated that *ARID1A* and *PIK3CA* mutations frequently coexist in both CCC (20–56%) [29] and EC (11–25%) [21,22]. In addition, Mabuchi et al. conducted proteome analysis of 98 primary ovarian tumors (52 CCCs and 46 HGSCs) using a tissue microarray and showed that AKT, mTORC1, and mTORC2 are more frequently activated in the former than in the latter [30]. PI3K/AKT/mTOR inhibitors have significant antitumor activity in ovarian cancer cells with high AKT/mTORC1 activity but only a slight effect in ovarian cancer cells with low AKT/mTORC1 activity [30,31]. Therefore, the PI3K/Akt/mTOR pathway has been highlighted as a biomarker for CCC therapy.

Genes encoding components of the mitogen-activated protein kinase (MAPK) pathway, such as *KRAS*, *PPP2R1A*, and *ERBB2*, are also frequently mutated in CCC and EC (Table 1 and Table 2). The MAPK pathway is involved in cell proliferation, survival, differentiation, and migration, and Itamochi et al. reported activation of this pathway in 20% of CCCs, with significantly higher overall survival (OS) in patients with MAPK pathway activation than in those without activation [16]. Unlike *PIK3CA* mutation, *KRAS* mutation does not necessarily coexist with *ARID1A* mutation. Therefore, the MAPK pathway and its downstream signaling pathways are also considered to be potential targets for cancer therapy in EAOC [32].

As mentioned above, the genomic profile of EAOC differs from that of HGSC, which is characterized by a high frequency of *TP53* mutation and homologous recombination deficiency (HRD), including *BRCA1* and *BRCA2* mutations (Table 1). Our previous study investigated germline and somatic mutations of 16 HR-associated genes in 207 ovarian cancer samples [33], and germline or somatic HR-associated gene mutations were detected in 44% of HGSCs but 28% of CCCs and 23% of ECs. In particular, the frequency of *BRCA1/2* somatic mutations was lower in CCC (5%) or EC (5%) than in HGSC (12%). On the other hand, the *ATM* gene, which encodes a regulator of the tumor-suppressor proteins p53 and BRCA1, checkpoint kinase CHK2, checkpoint proteins RAD17 and RAD9, and DNA repair protein NBS1, is more frequently mutated in CCC (9%) and EC (18%) than in HGSC (4%). Because ATM plays a key role in the DNA damage response [34], its inactivation is associated with high sensitivity to the PARP inhibitor olaparib in mantle cell lymphoma [35], colorectal cancer [15], and prostate cancer [36]. Therefore, PARP inhibitors may provide a survival advantage for those with *ATM*-mutated CCC or EC.

Copy number alterations are also important genomic events related to cancer [37]. It has been reported that the frequency of copy number alterations is much lower in CCC than in HGSC [38] and that the ratio of whole-arm copy number alterations is significantly higher in CCC [39]. Amplification of chromosome 8 is detected in 52% of CCCs [39], especially *ZNF217* copy number gain in CCCs, which is also frequently detected (in 20–36%) of CCCs [39,40,41]. Interestingly, *ZNF217* amplification is associated with clinically aggressive behavior such as recurrence or metastasis in CCC [40].

Furthermore, *MET* (chr7q31) (31%) and *AKT2* (chr19q13.2) (24%) are amplified in 31% and 24% of CCCs, respectively [42]. Copy number loss at *CDKN2A*/*2B* (9p21.3) is also often found [38]. Although these copy number alterations are potential molecular targets, a new treatment strategy based on copy number alterations has not been developed for CCC [43]. On the other hand, whole-exome sequencing studies have identified some EC samples with widespread copy number alterations, similar to the genomic instability demonstrated by HGSC [20,21,22]. As an example, Hollis et al. reported that the copy number alteration burden of EC varies by molecular subgroup defined by the mutation status of *TP53* and *CTNNB* [22]. These authors showed that cases of *TP53* mutation (26%) harbored a greater copy number alteration burden than cases of wild-type *TP53* (74%). In addition, they reported that *CTNNB* mutation cases with wild-type *TP53* had a lower copy number alteration burden than wild-type *CTNNB* cases with wild-type *TP53*. Comparing the clinical behavior of the above three genomic subtypes of EC, the group with a high copy number alteration burden (*TP53* mutation group) had worse outcomes, whereas the group with the lowest burden (*CTNNB1* mutation group with wild-type *TP53*) had favorable outcomes [22]. 

In general, it is difficult to distinguish high-grade EC from HGSC (WHO classification of tumors of the female genital tract) [44]. Indeed, common genomic alterations such as *TP53* mutations and copy number alterations were observed in both high-grade EC and uterine corpus serous. So-called “high-grade” endometrial carcinomas show worse prognosis [20,22,45]. Instead of genomic differences, Assem et al. examined six immunohistochemical markers (PAX8, WT1, p53, CSKN2A, dMMR, ARID1A) in grade 1–3 EC and high-grade serous ovarian carcinoma samples [46]. Corresponding to genomic results, more grade 3 ECs showed abnormal p53 expression compared with grade 1 ECs. Parra-Herran et al. demonstrated that MMR and *POLE* alterations were identified in a subset of ovarian endometrioid carcinoma with excellent prognosis [47].

Liquid biopsies of circulating tumor DNA (ctDNA) have recently been attracting attention as a non-invasive diagnostic and monitoring tool for solid cancers [48,49]. In s previous study, liquid biopsies from ctDNA and target sequencing were performed in 51 ovarian cancer patients with different histological subtypes [50]. In CCC, *APC* and *DCAF12L1* were mutated preferentially (30.8%), followed by mutations in *TP53*, *PIK3CA*, and *PDGFRA* mutations (23.1%). In EC, *PIK3CA* and *SLITRK5* mutations were detected frequently (40%). Higher cell-free (cf) DNA concentration significantly correlated with worse progression-free survival (PFS) in all patients. Further, patients with any pathogenic mutations showed significantly worse PFS. These findings suggested that ctDNA-based gene profiling might be used for the prediction of prognosis and planning the therapeutic strategies.

## 3. Cancer-Associated Gene Mutations in Endometriosis

Genomic studies of endometriosis were very limited before the advent of next-generation sequencing. Sato et al. showed that 13 of 23 ovarian endometriosis cases (56.5%) displayed loss of heterozygosity (LOH) of *PTEN* [51], suggesting that *PTEN* inactivation is an early event in the development of malignant transformation of ovarian endometriosis. In 2010, Wiegand et al. not only found that CCCs frequently harbor *ARID1A* mutations but also reported that in a limited number of cases, *ARID1A* mutations and ARID1A loss occur in CCC and contiguous atypical endometriosis but not in distal endometriosis [6]. Subsequent studies using immunohistochemistry have shown that ARID1A expression is usually lost in endometriosis coexisting with CCC, corresponding to the ARID1A expression level in carcinoma, but that ARID1A expression is preserved in distal or benign endometriosis [52,53].

More recently, genomic analysis based on next-generation sequencing has revealed the presence of cancer-associated gene mutations in endometriosis [12,13,14]. A list of representative cancer-associated gene mutations detected in endometriosis is shown in Table 3. We sequenced 107 ovarian endometrial epithelium samples (13: whole-exome sequencing and 94: targeted gene sequencing) and identified many cancer-associated gene mutations that are frequently identified in EAOC. For instance, we clarified that *KRAS* and *PIK3CA* followed by *ARID1A* were the most frequently mutated genes with high mutant allele frequencies (MAFs). In addition, we performed multiregional sampling of several sites from the same case and identified that cancer-associated gene mutations were homogeneously present in all sampling sites of the ovarian endometriotic cyst. Thus, cancer-associated gene mutations may induce endometriotic epithelial cells to expand clonally in the ovary.

Anglesio et al. performed genomic analysis of deep-infiltrating endometriosis (DIE), which is a rare and highly invasive form of endometriosis that infiltrates within extrauterine organs such as the uterosacral ligaments or colon [13]. Whole-exome sequencing revealed 80 somatic mutations in 19 of 24 patients (79%) with DIE, with five harboring oncogenic driver mutations, such as in *ARID1A*, *KRAS*, *PIK3CA*, and *PPP2R1A*. In an independent dataset, targeted gene sequencing and droplet digital polymerase chain reaction (PCR) identified *KRAS* mutations in two of three patients and three of 12 patients, respectively, and 10 DIE patients (26%) harbored at least one oncogenic driver mutation. Anglesio et al. also performed targeted gene sequencing in incisional endometriosis (IE), an iatrogenic form of endometriosis that occurs in obstetrics or gynecological operation scars [14], but *KRAS* and *PIK3CA* mutations occur less frequently in IE (5% and 2.5%).

The above three studies demonstrate that endometriosis harbors cancer-associated gene mutations regardless of the site of the lesion. Interestingly, *KRAS* mutation is the most frequent event occurring in ovarian endometriosis, DE, and IE (Table 3). Although 43% of ovarian endometriosis cases harbor *KRAS* mutations, the frequency of malignant transformation of ovarian endometriosis is 0.5–1% [54]. Given the lower frequency of *KRAS* mutation in EAOC compared to endometriosis, it is possible that *KRAS* mutation plays a different role than malignant transformation of endometriosis. For instance, *KRAS* mutation is involved in antiapoptotic effects called oncogene-induced senescence [55] and may contribute to the survival of endometriotic epithelial cells in ovarian endometriotic cysts, which are harsh environments with hemolysis and elevated levels of free heme and iron [56,57]. This hypothesis is consistent with the findings that *KRAS*-mutated cells are able to clonally expand inside ovarian endometriosis [12]. Moreover, specific types of *KRAS* mutations, such as *KRAS* p.G12D or p.G12V, are associated with inflammation [58,59,60]. Further studies are needed to clarify the biological significance of cancer-associated gene mutations in endometriosis.

## 4. Mutational Profiling of the Normal Uterine Endometrium

Prior to the era of next-generation sequencing, Mutter et al. reported loss of PTEN expression in the normal endometrium in 43% (24/56) of cases [61]. PTEN loss occurs in an entire endometrial gland rather than in a single cell, suggesting that endometrial glands are monoclonal units. Although it has long been difficult to identify somatic mutations with low frequency in a few cells of normal tissues [62,63], next-generation sequencing technology has in the last few years enabled the identification of mutations of oncogenes and tumor suppressors in histologically normal skin [64], the esophagus [65], colon [66], liver [67], bladder [68], and endometrium [12,69,70]. Table 4 lists cancer-associated gene mutations in the normal endometrial epithelium. In our previous study, we performed whole-exome sequencing of 11 endometrial epithelium samples collected by laser microdissection [12] and identified cancer-associated mutations, such as in *PIK3CA* and *KRAS*, with low MAFs. At the same time, we isolated 109 single epithelial glands from three cases and performed targeted sequencing for 84 cancer-associated genes. Somatic mutations in cancer-associated genes such as *PIK3CA*, *KRAS*, *FBXW7*, *PPP2R1A*, *PIK3R1*, and *ARID1A* occurred in a nearly clonal state characterized by high MAFs, supporting that endometrial glands are monoclonal units. When combined with the findings that *KRAS*- or *PIK3CA*-mutated cells expand clonally in endometriosis, *KRAS*- or *PIK3CA*-mutated cells may contribute to the implantation and survival of endometrial cells at ectopic sites after retrograde menstruation.

The frequencies of RAS/MAPK and PI3K/AKT pathway alterations identified by next-generation sequencing were compared among uterine endometrium [69], ovarian endometriosis [12], CCC [16], and EC [22]. 

Lac et al. performed target sequencing for normal endometrium in 25 hysterectomy and 85 curettage or biopsy specimens [70] and found that 54% of cases carried cancer-associated mutations. *KRAS* and *PIK3CA* were frequently mutated, whereas *ARID1A* mutations were not observed in the normal endometrium. The authors investigated the relationship between the prevalence of cancer-associated mutations and aging in the normal endometrium and found that the risk of harboring somatic mutations increased by 5% per year.

Additionally, Moore et al. performed whole-genome sequencing of 292 endometrial glands collected from 28 cases by laser microdissection [69], with 25 of the 28 (89.3%) cases harboring somatic cancer-associated gene mutations. Corresponding to our results, *PIK3CA* was the most frequently mutated (over 50% of the cases), and somatic mutation MAFs ranged from 0.3–0.5, supporting that endometrial glands are monoclonal units. The researchers also observed a linear accumulation of 29 base pair mutations per gland per year and concluded that the mutation burden increased with aging, as reported for other normal tissues [62]. Moreover, the normal endometrium exhibited significantly lower mutation loads for base substitutions and indels compared to endometrial cancers and showed no *POLE* mutations, deficiency in DNA mismatch repair genes, or copy number alterations, which are molecular characteristics in endometrial cancer. Driver genes of endometrial carcinoma, such as *PTEN, CTCF*, *CTNNB1*, *ARID1A*, and *TP53,* were also less frequently mutated in the normal endometrium.

## 5. Genomic Linkage from the Uterine Endometrium to Endometriosis and EAOC

Molecular analysis of the similarities between carcinoma and contiguous or concurrent endometriosis has been performed. Sato et al. showed that contiguous endometriosis had the same mutational or LOH status for *PTEN* as coexisting CCC or EC and suggested that *PTEN* inactivation occurred in the early stage of carcinogenesis [51]. Wiegand et al. demonstrated that CCC and contiguous endometriosis share the same *ARID1A* mutation and loss of ARID1A expression [6]. Other studies have confirmed that ARIDA loss occurs in atypical endometriosis [52,71,72,73]. Corresponding to pathological findings, atypical endometriosis is considered to be a precursor lesion of CCC or EC at the genomic level. Yamamoto et al. performed Sanger sequencing and identified similar *PIK3CA* mutation profiles between endometriosis without atypia and coexisting cancer [5]. Nonetheless, most of the studies described above focused on a single gene, and how EAOC evolves from endometriosis was not discussed. Anglesio et al. used next-generation sequencing technology to identify mutation profiles for CCC and concurrent endometriosis and found shared *ARID1A* and *PIK3CA* mutations [74]. In addition, some concurrent endometriotic lesions harbor most of the somatic mutations detected in primary CCC, and ancestral mutations have been identified in not only cancer-adjacent but also in tumor-distant endometriotic lesions. These findings provide evidence that endometriotic cells with cancer-associated gene mutations have already expanded in concurrent ovarian endometriosis which is the precursor of CCC.

Cancer-associated gene mutations are identified in both endometriosis [12,13,14] and normal endometrium [12,74,75]. By multiregional sequencing, we demonstrated that epithelial cells with cancer-associated mutations such as oncogenic *PIK3CA* and *KRAS* mutations clonally expand in ovarian endometriosis without cancer [12]. Our single-endometrial gland sequencing found that each gland carries distinct cancer-associated gene mutations, demonstrating the heterogeneity of the genomic architecture of the uterine endometrial epithelium [12]. These findings support Dr. Sampson’s retrograde menstruation hypothesis, whereby endometriosis derives from menstrual dissemination of endometrial tissue into the peritoneal cavity at the genomic level.

**Figure 1:**
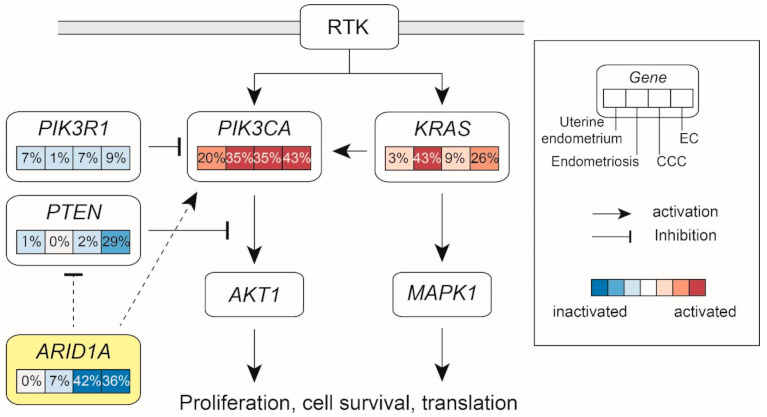
Alteration of PI3K/AKT and RAS/MAPK pathways in endometrium, endometriosis, and endometriosis-associated ovarian cancer (EAOC).

Figure 1 shows that the frequencies of RAS/MAPK and phosphatidylinositol-3-kinase (PI3K)/AKT pathway alterations identified by next-generation sequencing were different among uterine endometrium [69], ovarian endometriosis [12], CCC [16], and EC [22]. Many genes related to RAS/MAPK or PI3K/AKT pathways are already mutated in endometriosis and normal endometrium. On the other hand, the frequency of *ARID1A* mutation is clearly higher in EAOC compared to endometriosis and normal endometrium, indicating that *ARID1A* mutation plays an important role in oncogenesis. Although *ARID1A* is not a direct component of the PI3K/AKT pathway, *ARID1A* mutation induces aberrant activation of the PI3K/AKT pathway, leading to increased cell proliferation and inhibition of apoptosis [75,76]. In a mouse model, coexistence of the loss of ARID1A expression and *PIK3CA* mutation is needed to promote ovarian and endometrial carcinogenesis [77,78], and these findings are consistent with high co-mutation rate of *ARID1A* and *PIK3CA* in clinical EAOC samples. 

Although nearly half of endometriosis samples harbor *KRAS* mutations, the frequency is less than 10% for normal endometrium and CCC (Figure 1). These results suggest that *KRAS* mutations in endometriosis do not necessarily contribute to carcinogenesis. This phenomenon can be explained by the concept of oncogene-induced cellular senescence [55]. In a mouse model harboring a conditional oncogenic *KRAS* G12V allele, multiple lung adenomas and a few lung carcinomas developed [79]. Senescence markers were highly expressed in adenomas but not in cancers. Lac et al. have suggested that early activation of *KRAS* induces senescence in endometrial cells and inhibits progression towards malignant transformation [70]. Furthermore, our recent study has reported that *KRAS* G12V mutant allele expression was associated with inflammation in ovarian endometriosis [80]. In other words, *KRAS* mutation alone may be insufficient to cause cancer. In general, at least three driver mutations are required for malignant transformation [81]. For example, in a conventional colorectal adenoma-to-carcinoma sequence, carcinogenesis begins with *APC* inactivation followed by genetic activation of an oncogene such as *KRAS* or *BRAF* or by genetic or epigenetic inactivation of a tumor suppressor gene such as *TP53* or *SMAD4* [82]. This concept can be applied to a carcinogenesis from normal endometrium to ovarian cancer, suggesting that not only oncogene mutations but also inactivation of tumor suppressor genes is needed for malignant transformation. 

**Figure 2:**
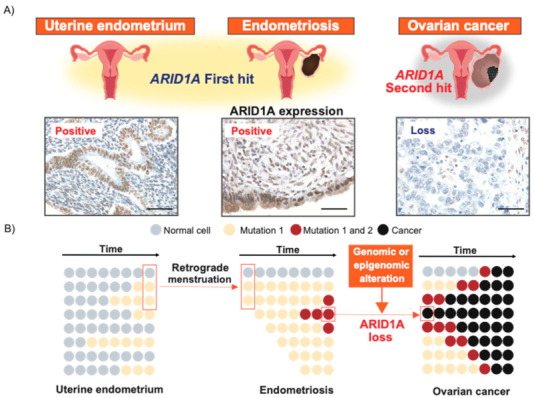
Molecular mechanism of EAOC development from uterine endometrium via endometriosis focused on *ARID1A*. (**A**) The second hit model of *ARID1A* in EAOC development from uterine endometrium via endometriosis. (**B**) The model that explain the process of *ARID1A* mutant cell progression for the development of EAOC.

However, there is no clear evidence to date that cancer develops from the endometrium via endometriosis. To provide evidence for a link between the uterine endometrium and EAOC through endometriosis, we performed whole-exome sequencing of the normal uterine endometrium, endometriosis, and cancer in a CCC patient [83]. Numerous somatic mutations, including cancer-associated mutations in *ARID1A*, *ATM*, *CDH4*, *NRAS*, and *PIK3CA,* were shared among the epithelium from the uterine endometrium, endometriotic lesions distant from and adjacent to the carcinoma, and the carcinoma itself. The MAF of the shared mutations increased from the uterine endometrium to the distant endometriosis and adjacent endometriosis sites, and the carcinoma. In particular, a splice site mutation of *ARID1A* was shared among the uterine endometrium, distant endometriosis, adjacent endometriosis, and clear cell carcinoma, whereas another frameshift mutation was specific to the adjacent endometriosis site and the carcinoma. Remarkably, these two types of mutations in *ARID1A* occurred in different alleles, suggesting that inactivation of *ARID1A* caused by a “second hit” [84] was the trigger of malignant transformation. To clarify the significance of ARID1A inactivation in the development of EAOC, we investigated whether ARID1A is expressed in ovarian endometriosis with an *ARID1A* loss-of-function mutation [53]. All *ARID1A* mutations identified in ovarian endometriosis were heterozygous mutations, and ARID1A protein expression was retained in all ovarian endometriosis samples despite loss-of-function mutations. On the other hand, the presence of *ARID1A* loss-of-function mutations was significantly associated with loss of ARID1A protein expression in CCC. In particular, ARID1A protein expression is absent in CCC samples that harbor multiple *ARID1A* loss-of-function mutations or *ARID1A* allelic imbalance. These results suggest that the second *ARID1A* hit is necessary for benign endometriosis with *ARID1A* heterozygous mutation to transform into cancer. Figure 2 shows the malignant transformation model of endometriosis focusing on *ARID1A* alteration. As inactivation of *ARID1A* is involved in epigenetic alterations and posttranscriptional and posttranslational modifications, both genomic and epigenetic or proteomic analyses are needed to search for a second hit of *ARID1A*.

Figure 2A. Uterine endometrium and endometriosis with and without *ARID1A* first hit retain ARID1A expression, while ovarian cancer with *ARID1A* second hit demonstrates loss of ARID1A expression.

Figure 2B. The image shows molecular mechanism of EAOC development from uterine endometrium via endometriosis. At first, endometrial epithelial cells with cancer-associated gene mutation in uterine endometrium transfer to ovary through a retrograde menstruation and demonstrate clonal expansion in endometriosis. Next, *ARID1A* heterozygous mutation occurs in the process of endometriosis development and second hit of *ARID1A* such as genomic or epigenetic alteration leads to malignant transformation of endometriosis. The illustrations were created with reference to Tomasetti et al. [85].

## 6. Conclusions

Driver gene mutations have been identified at the genetic level in benign endometriosis, which is the origin of endometriosis-associated ovarian cancer, as well as in the normal endometrium, which is the origin of endometriosis. The next issue is to clarify a trigger of carcinogenesis based on genomic lineage from normal endometrium to EAOC via endometriosis. To address these issues, multiomics analysis combining genomics, transcriptomics, and proteomics will be necessary. In other words, we are entering the postgenomic analysis stage. Further research on the normal endometrium and endometriosis may lead to the prevention of endometriosis and ovarian cancer development.

## Figures and Tables

**Table 1:** Frequency of somatic gene alterations in ovarian clear cell carcinoma.

Authors [Ref]	No of Samples	*ARID1A*	*PIK3CA*	*PPP2R1A*	*ARID1B*	*KRAS*	*PIK3R1*	*TP53*	*PTEN*
Wang et al., 2017 [15]	35	19 (54%)	33 (51%)	6 (17%)	NA	3 (9%)	3 (9%)	2 (6%)	2 (6%)
Itamochi et al., 2017 [16]	55	23 (42%)	19 (35%)	NA	10 (18%)	5 (9%)	4 (7%)	NA	1 (2%)
Murakami et al., 2017 [17]	39	24 (62%)	20 (51%)	4 (10%)	4 (10%)	4 (10%)	3 (8%)	NA	2 (5%)
Shibuya et al., 2018 [18]	48	32 (67%)	24 (50%)	9 (19%)	4 (8%)	8 (17%)	5(10%)	2 (4%)	1 (2%)
Kim et al., 2018 [19]	15	6 (40%)	6 (40%)	3 (20%)	NA	3 (20%)	NA	2 (13%)	2 (13%)
Total of frequencies	192	104 (54%)	102 (53%)	22 (16%)	18 (13%)	23 (12%)	15 (8%)	6 (6%)	8 (4%)

**Table 2:** Frequency of somatic gene alterations in ovarian endometrioid carcinoma.

Authors [Ref]	No of Samples	*PIK3CA*	*CTNNB1*	*PTEN*	*ARID1A*	*KRAS*	*TP53*	*PIK3R1*
Wang et al., 2017 [15]	30	15 (50%)	9 (30%)	NA	12 (40%)	11 (37%)	8 (27%)	2 (7%)
Cybulska et al., 2019 [20]	36 *	14 (39%)	9 (25%)	12 (33%)	7 (19%)	15 (42%)	6 (17%)	4 (11%)
Pierson et al., 2020 [21]	26	7 (27%)	11 (42%)	12 (46%)	5 (19%)	6 (23%)	5 (19%)	5 (19%)
Hollis et al., 2020 [22]	112	48 (43%)	54 (48%)	32 (29%)	40 (36%)	29 (26%)	29 (26%)	10 (9%)
Total of frequencies	234	84 (41%)	83 (41%)	56 (32%)	64 (31%)	61 (30%)	48 (24%)	21 (10%)

* Whole-exome sequencing (28 samples) and target sequencing (8 samples).

**Table 3:** Summary of genomic alterations in endometriosis.

Study [Ref]	Site of Endometriosis	No of Samples for Each Sequencing Method	Material	Method of Collection Samples	High-Frequency Cancer-Associated Gene Mutations (Proportion of Samples)
Anglesio et al., 2017 [13]	Deep infiltrating endometriosis	Total no of samples: 39 24: Whole-exome sequencing 3: Target gene sequencing 12: droplet digital PCR	FFPE (Formalin fixed paraffin embedded)	LMD (Laser Microdissection) or macrodissection	*KRAS* (15%) *ARID1A* (5%) *PIK3CA* (3%) *PPP2R1A* (3%)
Suda et al., 2018 [12]	Ovarian endometriosis	Total no of samples: 107 13: Whole-exome sequencing 94: Target gene sequencing	Frozen section	LMD	*KRAS* (43%) *PIK3CA* (35%) *ARID1A* (7%) *TAF1* (7%) *FAT1* (5%) *ARHGAP35* (4%) *PPP2R1A* (1%)
Lac et al., 2019 [14]	Incisional endometriosis	Total no of samples: 40 40: Target gene sequencing	FFPE	LMD or macrodissection	*KRAS* (5%) *PIK3CA* (2.5%) *ERBB2* (2.5%)

**Table 4:** Summary of genomic alterations in uterine endometrium.

Study [Ref]	No of Glands or Samples for Each Sequencing Method	Material	Method of Collection Samples	Age	High-Frequency Cancer-Associated Gene Mutations (Proportion of Samples or Glands)
Suda et al., 2018 [12]	11 samples (11 patients): Whole-exome sequencing	Frozen section	LMD	38–57 (median: 46)	*ARHGAP35* (33%) *PIK3CA* (32%) *PIK3R1* (17%) *FBXW7* (12%) *PPP2R1A* (7%) *KRAS* (13%) *PTEN* (4%)
	71 samples (29 patients): Target gene sequencing				
	109 glands (3 patients): Target gene sequencing	Single glands		38,47,49	*PIK3CA* (27%) *ARHGAP35* (12%) *PIK3R1* (11%) *FBXW7* (9%) *PPP2R1A* (8%) *KRAS* (5%) *ARID1A* (2%)
Lac et al., 2019 [70]	25 samples (25 patients): Target sequencing	FFPE	Macrodissection	21–61 (median: 37)	*KRAS* (35%) *PIK3CA* (13%) *FGFR2* (5%) *NRAS* (2%) *AKT1* (1%) *ERBB2* (1%) *PTEN* (1%)
	85 samples (85 patients): Target sequencing	Curettage or biopsy	NA		
Moore et al., 2020 [69]	257 glands (28 patients): Whole-genome sequencing	FFPE or frozen section	LMD	19–81 (median: 39)	*PIK3CA* (20%) *ARHGAP35* (7%) *PIK3R1* (7%) *FBXW7* (5%) *ZFHX3* (4%) *CHD4* (4%) *FOXA2* (4%) *KRAS* (3%)
